# Case of Poisoning from Opium, Wherein Read's Stomach-Pump Was Successfully Employed

**Published:** 1825-08

**Authors:** J. Ashford

**Affiliations:** Surgeon, &c.


ART. VIII.—***Case of Poisoning from Opium, wherein Read's Stomach-Pump was successfully employed.*** By J. ASHFORD, Esq. Surgeon, &c.

HAVING lately succeeded, by the use of "Read's stomach-pump," in a case where laudanum had been taken with the intent to poison, I am induced to send an account of it to the Medical and Physical Journal. Although the utility of this instrument is established beyond doubt, as yet it may be said to be new; so that every additional fact of its success will be of service, thereby tending to its early adoption in like cases; and as an inducement for practitioners in general to provide themselves with this most simple and effectual apparatus for removing poison from the stomach.

On the 30th of May last, I was requested to see Elizabeth Sandys, who, I was informed, was ill, in consequence of having, a short time previous, swallowed a quantity of laudanum, with opium together. Being away from home when I was first sent for, and as no time should be lost, a scruple of the sulphate of zinc was given, and the dose repeated in about ten minutes.

The time of my first seeing her was about twenty minutes from the first dose of the above medicine, and about threequarters of an hour from the time of swallowing the poison. There appeared no disposition to vomit, and the effects of the opium were sensibly increasing. I then mixed two tea-spoonsful of mustard (as is prepared for domestic purposes), with four ounces of tepid water, as the readiest method of producing vomiting, which, under all circumstances, I was disposed to believe would effect the removal of the deleterious drug from the stomach. Very shortly after this, the patient vomited once, and plentifully: she appeared much relieved. I waited for some time, and, as the symptoms did not then indicate the necessity for further interference, I left, directing them to suffer the patient to lie down, and, should there appear necessity, to send for me again.

About an hour after, I was desired to see her, as she had "gone fast asleep, the face becoming almost black, frothing at the mouth, and snoring very loud." These were the expressions used. As there remained this only remedy, I began the use of the syringe, and injected about three quarts of warm water into the stomach. The irritation of the fauces, produced by the introduction of the tube, disposed the stomach to get rid of its contents. This it partly did; and the rest was evacuated by means of the syringe. When nearly the whole of the fluid had been returned, there came the remaining part of the mustard emetic; and after this a dark-brown mucus, which I supposed to be the least unmixed portion of the laudanum. This I could not detect by the smell, as the mustard had wholly deprived the opium of its peculiar odour.

Having, as I supposed, done all possible, as far as related to the removal of the poison from the stomach, by this mean, my next object was to clear the intestines: this required strong and repeated doses, both of drastic and saline purgatives. The patient was not suffered to sleep until the cathartics had freely acted upon the bowels. Both before this was effected and for some time after, it was with difficulty she could be kept awake.

As might have been expected, fever followed, accompanied with hysterical paroxysms, which in a few days was subdued by usual remedies, and the patient ultimately recovered. I have since ascertained that between two and three ounces of tincture of opium, and about half a scruple of crude opium, was the quantity taken.

*Hinckley; 7th June,* 1825.

